# Artificial intelligence tools to assess different levels of activity performed by semi-wild horses in grassland ecosystems

**DOI:** 10.1007/s10661-025-14363-1

**Published:** 2025-07-16

**Authors:** Anna Chodkiewicz, Martyna Prończuk, Marcin Studnicki

**Affiliations:** 1 Department of Agronomy, Institute of Agriculture, Warsaw University of Life Sciences, 159 Nowoursynowska Str., 02-776 Warsaw, Poland; 2 Warsaw, Poland; 3 Department of Biometry, Institute of Agriculture, Warsaw University of Life Sciences, 159 Nowoursynowska Str., 02-776 Warsaw, Poland

**Keywords:** Accelerometer, CART method, Horse behaviour, Machine learning

## Abstract

In order to understand the role of horses in ecosystems and to effectively use their grazing in the protection of grasslands, it is important to assess where they primarily stay, followed by whether these habitats are used for grazing or resting. The main goal of the study was the model development based on artificial intelligence tools which allow to distinguish the basic levels of activity performed by horses using data from an accelerometer mounted in a collar worn by animals. The model calibration was based on direct observations of five randomly selected Polish primitive horse mares. In order to create a model that allows for classification into three groups of behaviours: grazing, resting, and moving, an approach based on machine learning, one of the basic technologies of artificial intelligence, was used. The carried out analyses allowed for the determination of the most important features, among the fourteen determined from raw *X*, *Y*, and *Z* axis acceleration values across 5-s measurements. The recommended method for the classification of behaviours of primitive Konik horses based on the selection of variables observed from the accelerometer is the CART method, whereas the most accurate tool for its construction is learning neural networks. Our research indicates the usefulness of the accelerometer and proposed artificial intelligence methods in distinguishing the main activities performed by horses.

## Introduction

Wild horses have historically been an essential part of European ecosystems. Currently, their grazing is one of the management tools to maintain biodiversity and open landscape (Metera et al., 2010). Among the horses used in nature conservation, primitive breeds such as Polish primitive horses (Konik horses; *Equus ferus caballus* Linnaeus, 1758) play a special role. In comparison to cattle, they have a greater ability to feed even on low-quality swards and lower liveweight, which favours their use in the protection of marginal areas such as wetlands (Menard et al., 2002; Prache et al., 1998). They are also usually characterised, among other things, by resistance to harsh environmental conditions, the ability to use low-quality forage, and the capability to compensate for periodic food shortages (Pasicka, 2013). The use of extensive Konik horses grazing in areas of natural value is popular not only in different regions of Poland, e.g. the Roztocze National Park, in Łąki Skoszewskie, a special protection area on the western coast of the Szczecin Lagoon, forming part of the Natura 2000 network as a Bird Refuge, Biebrza National Park (Chodkiewicz, 2020) but also in other European countries like France (Moindardeau et al., 2021), England (Stroh et al., 2012), the Netherlands (Vulink, 2001), Belgium (Cosyns et al., 2001), Germany (Köhler et al., 2016), and Latvia (Reķe et al., 2019). The breed is being successfully utilised in the protection of different habitats, including heaths, coastal dunes, calcareous swards, or in marshlands (Chodkiewicz, 2020). Part of the Konik horses’ population is kept in the reserve breeding, where the animals stay in natural conditions with as little human intervention as possible, limited to herd population control or hay provision in the food shortage period (Polish Horse Breeders Association, 2020).

In the use of extensive grazing of horses in protected areas as part of active habitat protection and in reserve breeding, where animals are often kept in large-scale enclosures (from several dozen to several hundred hectares), the problem is to monitor their activity and time spent grazing in different habitats. This is important both for the assessment of animal welfare and the fulfilment of the objectives of the activities carried out (grazing efficiency and the condition of grazing habitats). Such knowledge should make it possible to assess whether there are areas potentially at risk of a negative impact of animals as a result of, e.g. overgrazing, trampling, or overfertilisation due to leaving dung or need additional action, like mowing, to achieve the conservation goal. Healthy, free-ranging horses satisfy their essential needs by allocating their time between grazing, rest, and movement, showing certain repeatable daily behaviour patterns (e.g. Berger et al., 1999). Most studies on the behaviour and habitat preferences of horses bred in reserves are based on direct observations of the animals (Auer et al., 2021). Such work is time-consuming, restricted for several consecutive days in selected months, and usually limited to specific time intervals during the day (e.g. Duncan, 1985; King, 2002). A negative impact of the observer’s presence on the horses’ behaviour could not be ruled out either.

Currently, in the monitoring of animals, including those of the family *Equidae*, collars equipped with GPS receivers are used, which, with a certain frequency, transmit information about their location, but they do not provide data on the levels of activity performed by animals (Kaczensky et al., 2008; Hampson et al., 2010; cf. Hennig et al., 2020). Due to that, the assessment of animal behaviour is still mainly based on direct observations. An alternative may be the use of cameras and the artificial intelligence programs to interpret the animals’ activity on pasture. Both methods have their constraints, e.g. observations/recordings are not always possible in dense vegetation, due to the large area of the enclosure or at night (Dunford et al., 2024; Riaboff et al., 2022). In this study, we used the GPS collars equipped with a GPS receiver and accelerometer. The usefulness of such devices in assessing the activity and utilisation of habitats by animals has been demonstrated so far, *inter alia*, for red deer (e.g. Schaefer et al., 2008), roe deer (Krop-Benesch et al., 2013), or cattle (e.g. Simanungkalit et al., 2021; Vázquez Diosdado et al., 2015). According to our knowledge, no studies have been performed to date on the behaviour and preferences of free- or limiting-ranging horses with the use of GPS transmitters equipped with an accelerometer. Therefore, the main goal of our research was to develop the model development which allows us to distinguish the different levels of activity performed by horses (that is grazing, resting, and movement) and can be used for long-term horses’ behaviour monitoring and for assessing their habitat preferences based on the data collected by using the remote sensing methods.

## Material and methods

### Study site

**Fig. 1:**
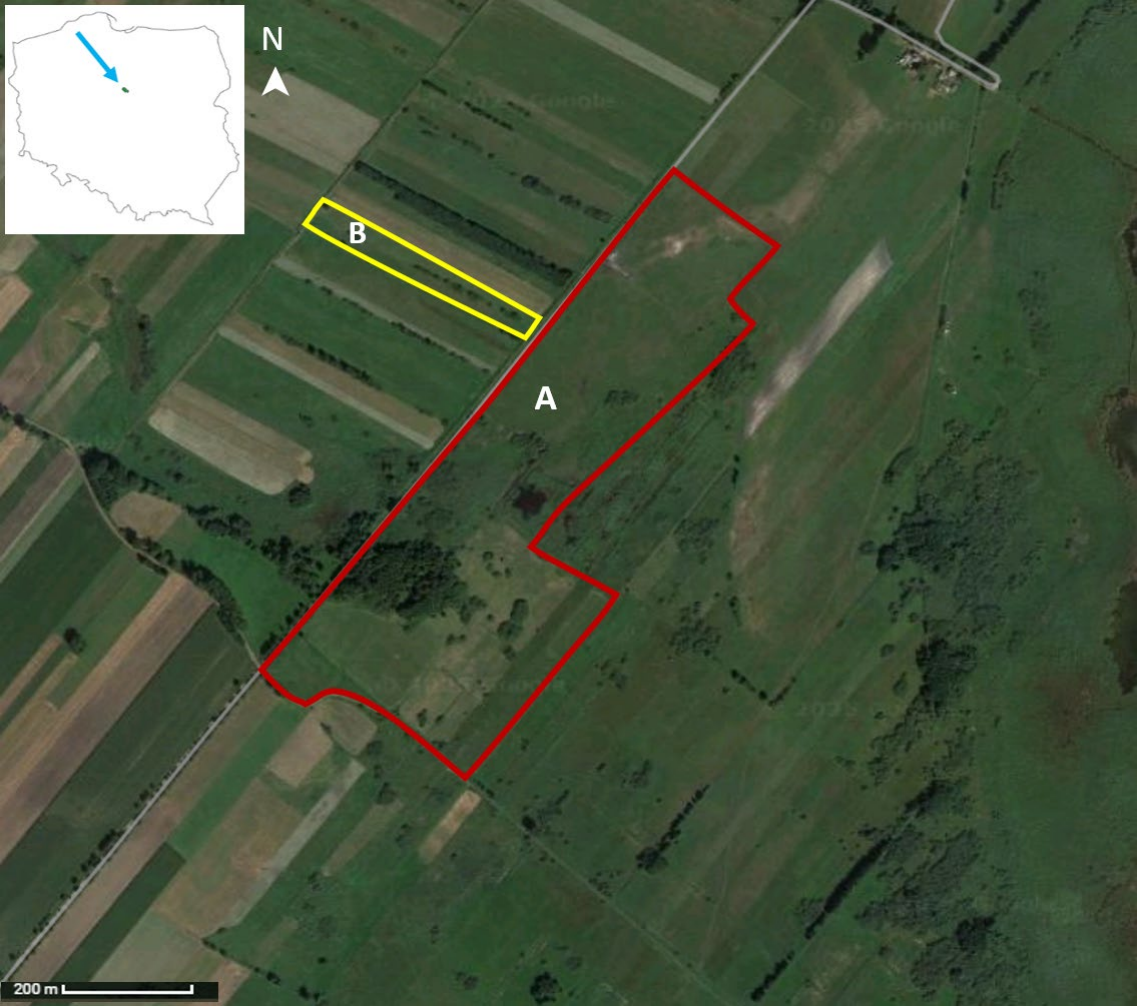
Study site. A and B enclosures in which horses were observed

The study was conducted in the Rakutowskie peatbogs (52.5373782521463, 19.21212531881468), which is a Natura 2000 nature protection area, located in the central part of Poland, within the Vistula Valley between the cities of Płock and Włocławek. In the centre of this region, the kettle and shallow Rakutowskie lake is surrounded by extensively used meadows and pastures, including areas of fen mires, reed and sedge meadows, and alder swamp forests. The duration of the growing season in this region is from 221 to 225 days (average from 1981 to 2010) (Krużel et al., 2015), whereas annual rainfall amounts range from 490 to 530 mm (Błażek et al., 2013). In 2015, the Society for Nature Alauda reintroduced extensive agriculture management on these lands and introduced a Konik horse herd. The animals spend the growing season (from April to November) on pastures without access to hay. The research was conducted when one herd of about 40 horses stayed alternately in two enclosures (A and B; Fig. 1) with an area of 30.7 ha and 1.38 ha, respectively. The animals were moved after reaching the target grazing point of the enclosures. During the study, the horses stayed in a smaller enclosure (B) twice (each time for about 2 weeks). The rest of the time, the animals spent in enclosure A. The communities dominant in the grazed area are *Molinion* meadows and fresh meadows with fragments of reeds. In each enclosure, horses also had access to willow thickets and/or grey alder trees.

### Horses observation

**Fig. 2:**
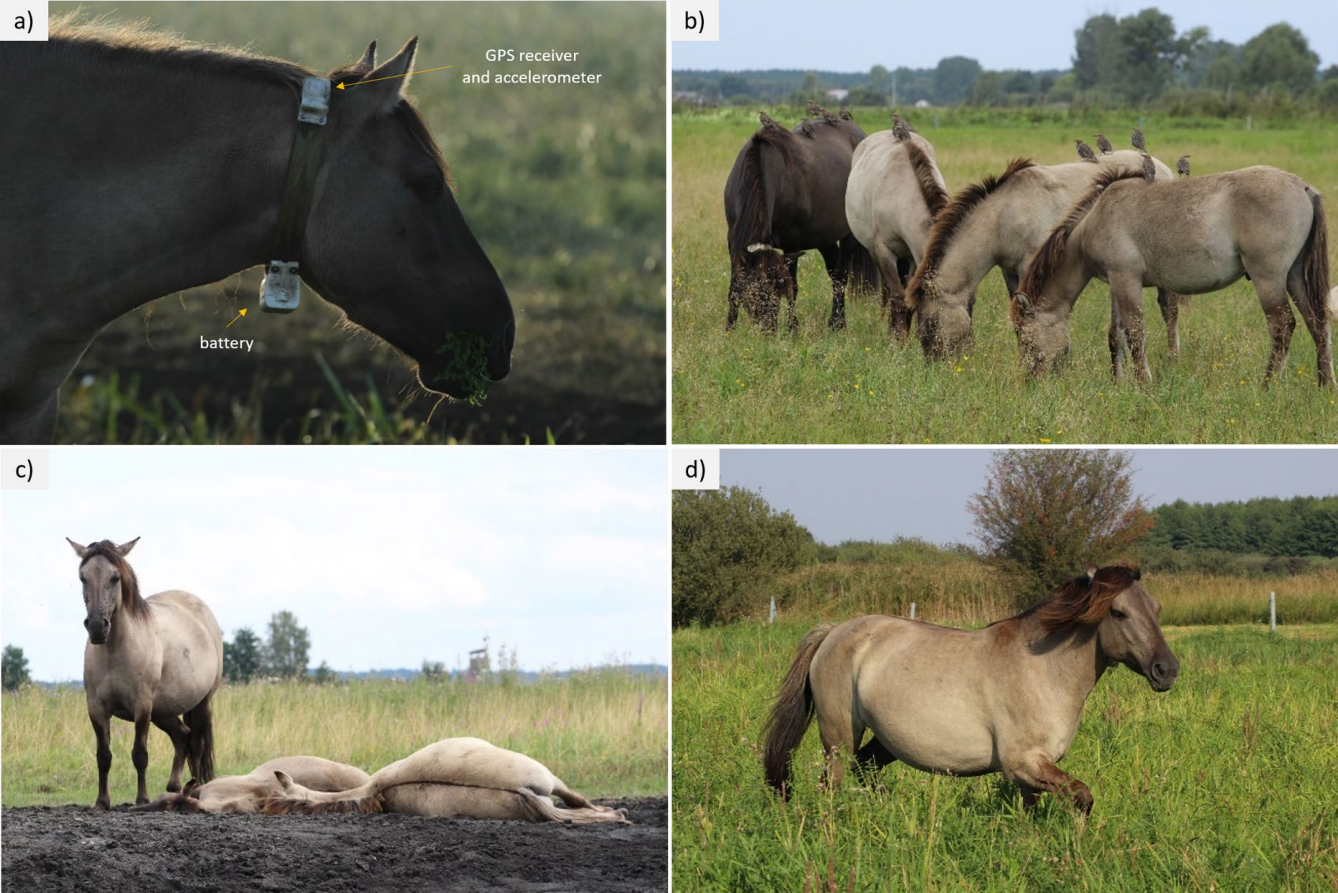
Horses on the pastures: **a** collar construction and different levels of activity performed by horses: **b** grazing, **c** resting in standing and lying position, and **d** moving (Phot. A. Chodkiewicz)

Direct observations of horse behaviour were conducted during the day in five terms in August and September 2024. The two collars equipped with GPS (ECOTONE) and an LSM6DSLTR (STMicroelectronics) accelerometer module were fitted to the healthy, randomly selected, not in foal mares a few days before the observations began. During each session, one mare wearing a collar equipped with a GPS and accelerometer module was observed independently of the weather. The observations were conducted by one observer on three consecutive days (observation time: minimum 6 h/day; on average, almost 7 h/day) in order to get the total observation time of 20 h per session/mare. One animal was observed in each term, so the total observation time of five different mares was 100 h. The height of Konik horses at the withers is on average 130–140 cm. The accelerometers were mounted on the neck of an animal to furnish information on its head movements by measuring the acceleration in a triaxial system (front-back, up-down, right-left; Fig. 2a). The accelerometer recorded data with a frequency of 5 Hz and a total of 32 measurements every 5 min (which will enable continuous measurement for 6 s); such a single measurement is called an epoch. The research was conducted from a distance (at least 10 m up to 30–40 m), also using binoculars, to limit the influence of human presence on the animals’ behaviour. During the observations, the horses’ behaviour (Fig. 2) was recorded with an accuracy of 1–3 s, with assignment to three categories: (1) grazing (the head is bent low to the ground, movements are made to break the sward), (2) resting in standing position (the horse stands still, relax, usually with its head raised, sporadic head movements are associated with chasing away insects) or lying (the horse lies motionless, the head is kept vertical or lying down), and (3) movement (quick mixing, head up). Other behaviours, including drinking (the horse’s posture is similar to grazing, is in short sequences, performed while watering holes), and including social behaviours: feeding foals, mutual scratching, scratching, playing, etc., were recorded, but due to their sporadic nature and small amount of collected data, they were not included in the analyses. There was no owner interference or any other treatments during observation time that could affect the horse’s behaviour. Accelerometer data downloaded from the server was then attributed to the behaviour. In total, 1081 accelerometer reading cycles (epoch) from observations of five different mares were collected.

### Behaviour classification model

In order to create a model that allows for classification into three groups of behaviours based on data for each of the 1081 epochs, an approach based on machine learning, one of the basic technologies of artificial intelligence, was used. The fourteen features were determined from raw *X*, *Y*, and *Z* axis acceleration values across 5-s measurements (Barwick et al., 2018, 2020). The following parameters were used:

- Minimum *X* axis (Min_X)—the minimum value for the *X* axis across one epoch
- Minimum *Y* axis (Min_Y)—the minimum value for the *Y* axis across one epoch
- Minimum *Z* axis (Min_Z)—the minimum value for the *Z* axis across one epoch
- Maximum *X* axis (Max_X)—the maximum value for the *X* axis across one epoch
- Maximum *Y* axis (Max_Y)—the maximum value for the *Y* axis across one epoch
- Maximum *X* axis (Max_Z)—the minimum value for the *Z* axis across one epoch
- Average *X* axis (A_X)—the averaged values for the *X* axis across one epoch, according to the formula:
  $$A\_X=\frac{1}{T}\sum\limits_{t=1}^{T}{X}_{t}$$
  where *T* is the total number of sample in epoch, and ${X}_{t}$ is the accelerometer of *X* axis in t sample in the epoch.
- Average *Y* axis (A_Y)—the averaged values for the *Y* axis across one epoch, according to the formula
  $$A\_Y=\frac{1}{T}\sum\limits_{t=1}^{T}{Y}_{t}$$
  where ${Y}_{t}$ is the accelerometer of *Y* axis in t sample in the epoch.
- Average Z axis (A_Z)—the averaged values for the *Z* axis across one epoch, according to the formula
  $$A\_Z=\frac{1}{T}\sum\limits_{t=1}^{T}{Z}_{t}$$
  where ${Z}_{t}$ is the accelerometer of *Z* axis in t sample in the epoch.
- Signal magnitude area (SMA)—the acceleration magnitude, summed across three axes within each epoch, is normalised by the epoch length (Zhang & Sawchuk, 2011), according to the formula:
  $$SMA=\frac{1}{T}\left(\sum\limits_{t=1}^{T}\left|{X}_{t}\right|+\sum\limits_{t=1}^{T}\left|{Y}_{t}\right|+\sum\limits_{t=1}^{T}\left|{Z}_{t}\right|\right)$$
- Movement variation (MV)—the overall variance within the signal epoch is assessed through a cumulative analysis of amplitude, frequency, and acceleration duration (Campbell et al., 2013), according to the formula:
  $$MV=\frac{1}{T}\left(\sum\limits_{t=1}^{T-1}\left|{X}_{t+1}-{X}_{t}\right|+\sum\limits_{t=1}^{T-1}\left|{Y}_{t+1}-{Y}_{t}\right|+\sum\limits_{t=1}^{T-1}\left|{Z}_{t+1}-{Z}_{t}\right|\right)$$
- Average movement intensity (AMI)—the average acceleration reading per sample throughout the epoch (Zhang & Sawchuk, 2011), according to the formula:
  $$AI=\frac{1}{T}\sum\limits_{t=1}^{T}{MI}_{t}$$
  where $${MI}_{t}=\sqrt{{\left|{X}_{t}\right|}^{2}+{\left|{Y}_{t}\right|}^{2}+{\left|{Z}_{t}\right|}^{2}}$$
- Entropy (S)—the predictability of the information content of a random variable is higher for time windows capturing smooth motion, which exhibit lower entropy, compared to windows capturing random motion, with entropy being a measure of “disorder” or freedom of motion (Alvarenga et al., 2016), according to the formula:
  $$S=\frac{1}{n}\sum(1+{Ts}_{i})\,\text{ln}\,(1+{Ts}_{i})$$
  where *n* is the number of records in the epoch and *Ts* = A_X + A_Y + A_Z.
- Energy (E)—the summing of squared signal components from each axis and normalising them by the epoch length, according to the formula:
  $$E=\frac{1}{n}\sum{Tss}_{i}^{2}$$
  where *Tss* = A_X^2^ + A_Y^2^ + A_Z.^2^

The data set of 1081 epochs with all above-presented variables was divided randomly into a training set and a test set in the proportion of 80:20.

The first data set used to create classification models will be based on principal component analysis (PCA), which will be performed for all 14 variables calculated from the accelerometer. PCA analysis allows for reducing the number of variables and eliminating information noise. The values of principal components whose eigenvalue is greater than 1 will be used as a data set. PCA was calculated only for the training data set.

The second data set used to create classification models will be based on raw data for selected variables from these fourteen that significantly affect the variability between behaviour categories. For this purpose, Pearson linear correlation coefficients were first used separately for each of the three types of behaviour. This will eliminate strongly correlated (greater than 0.95, regardless of the sign) variables that may contribute to the occurrence of the phenomenon of collinearity. After excluding strongly correlated variables from the set, the next step was to use the classification and regression trees (CART) method to select those specific parameters calculated from the accelerometer data that significantly affect the classification into these three behaviour groups. The CART method selects significant variables in the classification using an approach based on the Gini Index that calculates the probability that a randomly selected case will be incorrectly classified. The following values of hyperparameters were indicated during the performance of these analyses: for the maximum depth, it was the value of 10; for the minimum number of observations that must exist in a split, it was equal to 15. Similarly to the PCA analysis, in this case, the correlation coefficients and the CART model were determined only on the basis of the training data set.

Both the data set based on the values of the principal components and the selected raw variables will be used to create a classification model based on machine learning techniques. The effectiveness of two groups of machine learning methods was applied and assessed—discriminant analysis and the approach based on learning neural networks. Three different discriminant analysis methods will be used:

- Linear discriminant analysis (LDA), which is a classic approach to classification,
- Quadratic discriminant analysis (QDA), which, unlike LDA, allows for independent estimation of the covariance matrix separately for each class (in our case, each behaviour),
- Mixture discriminant analysis (MDA) allows for independent assumptions of multiple normal distributions for individual classes and subclasses.

During the approach based on learning the neural network, we used the neuralnet package of the R software. Due to the relatively small number of observations, we used two hidden layers, each with 5 neurons. In total, we used 4 different machine-learning methods. The assessment of the precision of the estimation of the proposed models will be based on the test set, and the prepared confusion matrix will determine parameters such as accuracy, precision, sensitivity, and specificity, according to equations (Alvarenga et al., 2016, Campbell et al. 2013):

- Accurecy = (TP + TN)/(TP + TN + FP + FN)
- Precision = TP/(TP + FP)
- Sensitivity = TP/(TP + FN)
- Specificity = TN/(TN + FN)

where TP (true positive) refers to the number of times the target behavioural class was accurately identified when it was actually present, FN (false negative) describes the instances in which the target behaviour occurred but was mistakenly labeled as a different class, FP (false positive) counts the situations where the model incorrectly predicted the presence of the target behaviour, even though it did not occur, and TN (true negative) represents the number of cases where the model correctly recognised that the target behaviour was not present.

Analyses and calculations were done using the R 4.4.2 software (R Core Team, 2025). The following packages were used: rpart for CART analysis, neuralnet for neural networks, MASS for LDA and QDA, and the mda package for MDA.

## Results

During the observation of the Konik horses’ behaviour, a total of 1081 epochs (observations) were obtained, of which 323 were classified as resting, which constituted 29.88% of all observations, 601 as grazing (55.60%), and 157 as movement (14.52%).

**Fig. 3:**
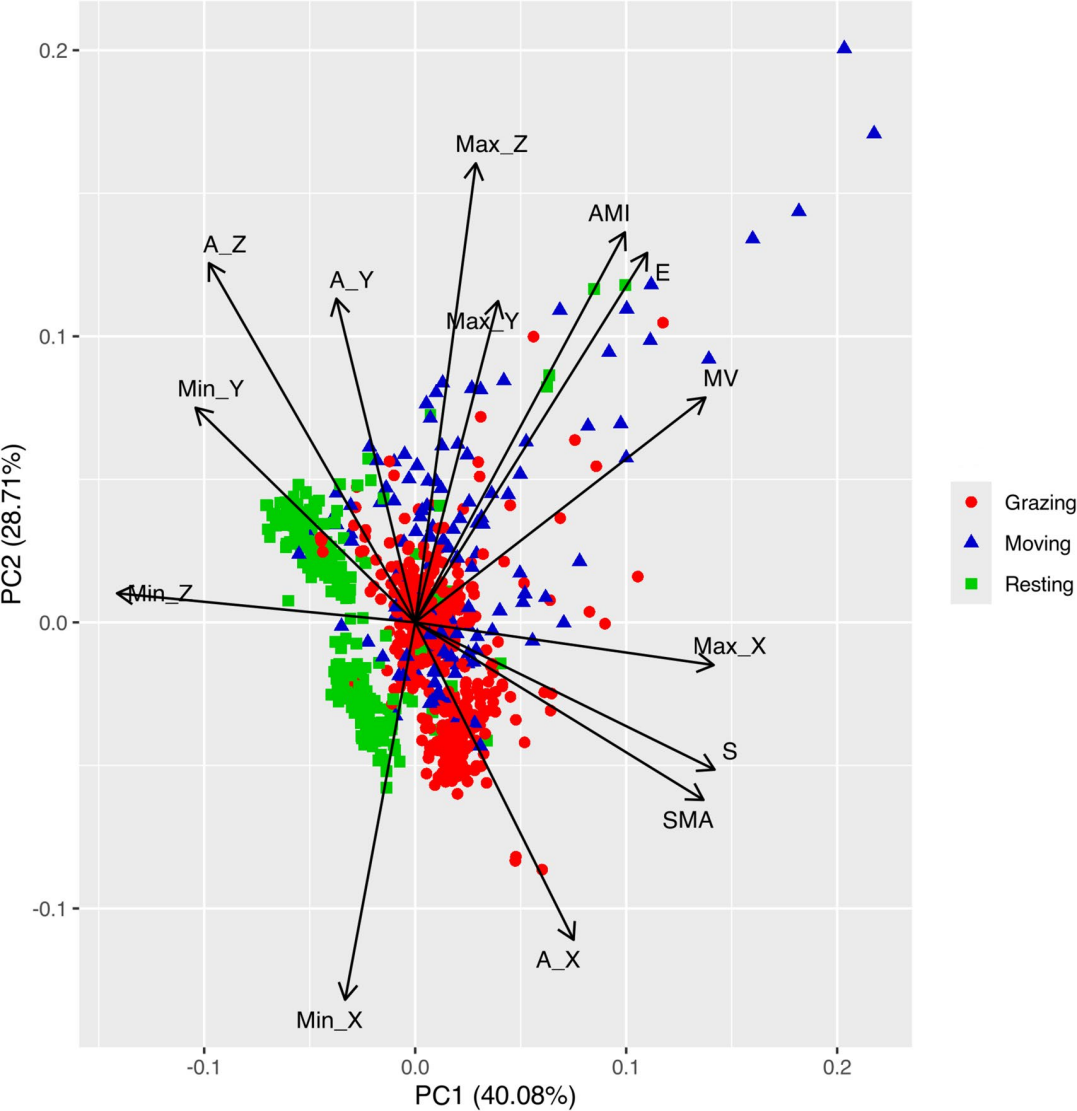
The biplot for principal component analysis (PCA) for all fourteen features accelerometer determined for the training data set

In Fig. 3, the principal component analysis (PCA) results are presented; the first two components explained more than 68% of the variability of the training data set based on the calculated 14 parameters. Among the epochs, those classified as motion were characterised by the highest variability for the first two components. Eigenvalues greater than 1 were observed for the first three components, which together explained about 86% of the total variability.

**Fig. 4:**
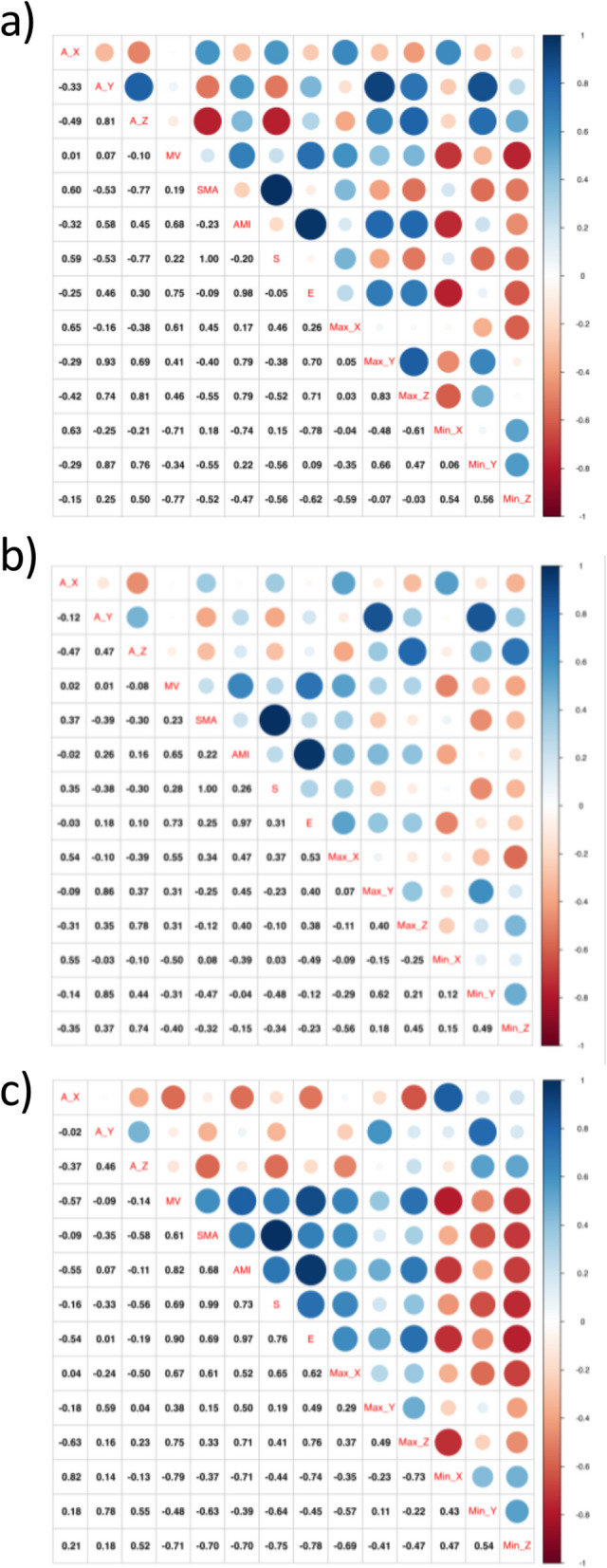
Correlation coefficient between all fourteen features determined by accelerometer from the training data set for behaviour: **a** resting, **b** grazing, and **c** moving

The correlation coefficients between all fourteen features determined by the accelerometer were presented for each behaviour separately in Fig. 4. In general, the weakest values of correlation coefficients are observed for grazing. Much stronger correlations are observed for the two remaining behaviour categories. A very strong positive correlation for each behaviour is observed between signal magnitude area (SMA) and entropy (S). A strong positive correlation is also observed between A_Y and Max_Y, but only for resting and grazing. Based on PCA analysis (Fig. 3) and regression analysis (Fig. 4), we observe two very strong correlations between SMA and entropy (S) and AMI and energy (E). Therefore, we gave up on the SMA and AMI parameters to further create the classification model, leaving entropy (S) and energy (E).

**Fig. 5:**
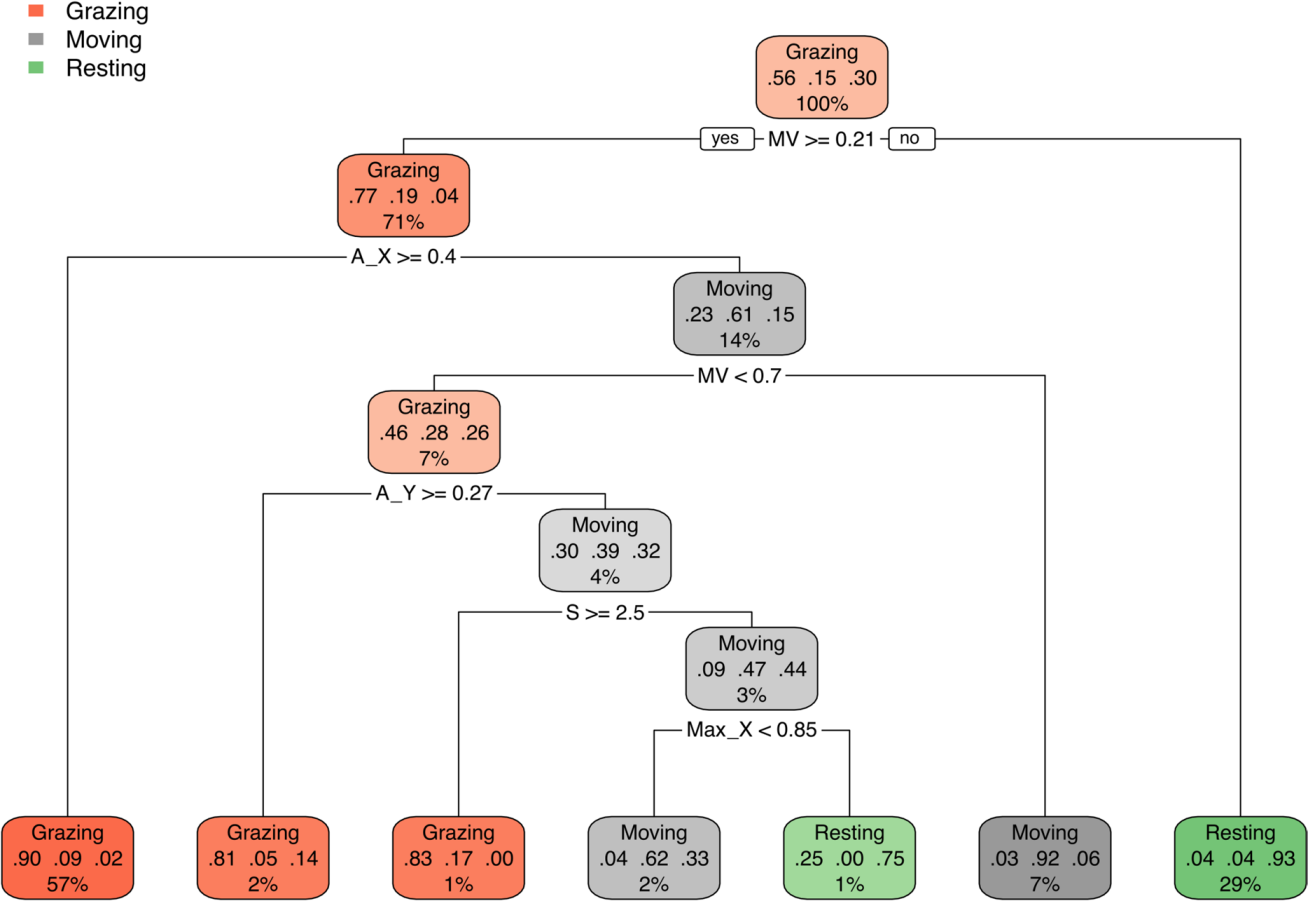
The classification and regression trees (CART) plot used for the selection of important features determined by the accelerometer

Figure 5 shows the results of classification and regression trees (CART) used to select variables of great importance in behaviour classification. Based on this analysis, we can indicate that the variables calculated based on the accelerometer information significantly impacting classification are A_X, Max_X, Max_Y, MV, and S. They will be used to build and compare models.

Table 1 presents general characteristics of classification accuracy for the applied models and two data sets created from 1081 epochs of accelerometer observations. Generally, we observe lower accuracy values for data composed of the first three principal components (PCA), meaning they have less accurate classification based on the confusion matrix. Higher values of this parameter are observed for the data set based on variable selection using CART. A similar effect is observed for the Kappa coefficient. The highest accuracy value is observed for the model created from learning neural networks for the data set created from selection based on CART. This combination of classification method and data set also had a Kappa coefficient value above 0.80, which may indicate high compliance with the model and test set classification.

**Table 1:** Parameters describing the fit of classification models based on two different data sets

Parameter	LDA	QDA	MDA	Neural network
a) A data set with raw features selected by CART				
Accuracy	0.8698	0.8558	0.8436	0.9123
95% CI	(0.8173, 0.9117)	(0.8016, 0.8999)	(0.8011, 0.8840)	(0.8564, 0.9454)
Kappa	0.7639	0.7464	0.7372	0.8034
b) Data set with PCA loadings				
Accuracy	0.8512	0.8372	0.8465	0.8915
95% CI	(0.7964, 0.8959)	(0.7809, 0.8839)	(0.7913, 0.8919)	(0.8214, 0.9251)
Kappa	0.7329	0.7081	0.7266	0.8005

The parameters of the classification accuracy assessment, separately for each behaviour, for the four models and both data sets, are presented in Table 2. In general, the highest classification errors are observed for movement among all three types of behaviour, as evidenced by the low values of the sensitivity parameter. The highest value of this parameter for movement is observed for the data set with selected variables using the CART method and the application of neural network training, where the sensitivity value was 0.4823.

**Table 2:** Parameters describing the fit of classification models created on the basis of two different data sets determined separately for each behaviour

Parameter	Data set with raw features selected by CART			Data set with PCA loadings		
	Grazing	Moving	Resting	Grazing	Moving	Resting
LDA						
Sensitivity	0.9667	0.3871	0.9219	0.9250	0.3548	0.9531
Specificity	0.8316	0.9946	0.9272	0.8211	0.9837	0.9205
Balanced accuracy	0.8991	0.6908	0.9245	0.8730	0.6693	0.9368
QDA						
Sensitivity	0.9333	0.4516	0.9062	0.9083	0.3226	0.9531
Specificity	0.8737	0.9511	0.9338	0.8105	0.9837	0.9073
Balanced accuracy	0.9035	0.7014	0.9200	0.8594	0.6531	0.9302
MDA						
Sensitivity	0.9667	0.3548	0.8906	0.9167	0.3871	0.9375
Specificity	0.8105	0.9946	0.9205	0.8211	0.9674	0.9338
Balanced accuracy	0.8886	0.6747	0.9056	0.8689	0.6772	0.9356
Neural network						
Sensitivity	0.9750	0.4823	0.9062	0.9333	0.4331	0.9323
Specificity	0.8316	0.9191	0.9272	0.8123	0.9320	0.9536
Balanced accuracy	0.9033	0.7608	0.9167	0.8542	0.7145	0.9534

## Discussion

Based on the results of our research, we can recommend a model for the classification of behaviours of primitive Konik horses based on the selection of variables observed from the accelerometer using the CART method and constructing a model based on learning neural networks. This approach had the best parameter values for classifying the three types of behaviours.

The proposed models did not differ significantly in efficiency, but among the 4 classification methods, the best one was based on neural networks. The research by Riaboff et al. (2022), a meta-analysis of research on classification models of farm animal behaviour, indicates that our models have the desired values of the indicated parameters, assessing the goodness of classification. The accuracy and the Kappa parameter values can be considered good, and our approach does not differ significantly from other model proposals.

Very often, in modelling and machine learning, variable reduction methods are often used, including the PCA method (Howley et al., 2006; Kleanthous, 2018). The PCA method allows for the simplification of multidimensional variables with minimal loss of important information. However, in our studies, using a data set based on values for individual principal components showed slightly worse results than raw data selected using the CART method.

Studies by Alvarenga et al. (2016) and Altenburg et al. (2021) show that the longer the duration of a single accelerometer measurement (epoch time), the better the models classify animal behaviour. In our studies, we did not attempt to assess the epoch time on the goodness of the models because epoch time extension would affect the observation time on one battery charge. Frequent battery replacement or collar charging would cause organisational problems (a difficult research area far from the scientific base), but we also want to limit human contact with animals.

None of the models determined were accurate in indicating the movement of the Konik horse; there may be several reasons, one of which may be the placement of the accelerometer on the animal’s neck. The variety of movement (walking, trotting, galloping) may also make it difficult to classify separately. As indicated by the research of Barwick et al. (2020), the position of the accelerometer on the animal had a significant impact on the efficiency of behaviour classification. Telemetry collars cannot be precisely fitted to the horse’s neck circumference, which results from the structure of its body. When the animal has its head raised, its neck circumference is the largest. A grazing animal tilts its head strongly; its neck circumference is smaller, and the collar moves towards the ears. The neck size of horses can also change during the seasons, e.g. expands as body condition improves in spring and summer when high-quality forage is available; thus, properly fitting, not too tight, the collar is important to prevent injuries (Collins et al., 2014). All this can contribute to additional vibrations and the slipping of the accelerometer. However, for practical reasons, placing the accelerometer in combination with the GPS receiver reduces the risk of it being lost or damaged during intense movement, while animals are rolling or, for example, when they break through dense forest/shrub vegetation. Previous research on accelerometer placement indicates that placing it on the neck on a collar is the most convenient and reliable. It also allows tracking the movements of the animal’s head while grazing (Hansen et al., 2007; Morrison et al., 2015). Wearing a collar is tolerated by horses and does not disturb their natural behaviour. In addition, the device has constant access to power and the ability to remotely transfer data. Another reason for the poor level of indicating the movement of the Konik horse is that our data set contained very few movement observation records. This makes our data set imbalanced across classes, as indicated by the research of Sakai et al. (2019); the class with fewer data points tends to be ignored. In our case, this may be the case with the class, which is movement.

In contrast to studies conducted on the use of accelerometers in stable husbandry (Morrison et al., 2015), where the main goal is to monitor the health of animals, detect early stages of laminitis, pain or minor injuries, the main aim of our study was to distinguish between grazing and resting. Direct observations of grazing animals indicate that horses spend up to 50–70% of their time (14–17 h) during the day grazing (Chodkiewicz, 2020). Intentional movement in the case of semi-wild Konik horses is usually limited to moving between individual habitats (mainly meadows) or places of grazing and resting. Its share in the horses’ time budget reaches 10%. Grazing sessions in horses are continuous; depending on the season and the nutritional value and height of the grazed vegetation, they can last 1–3 h. Similarly, the rest time is usually 1–2 h (own observation). In reserve breeding, horses have access to a variety of forest and meadow communities. In the context of using grazing as a tool for active conservation, the most important thing is to assess where horses primarily stay, followed by whether these habitats are used for grazing or resting. The latter will be related to the intensity and nature of the animals’ impact on vegetation. In resting places, horses concentrate on a small area, leaving larger amounts of dung, often lying down and rolling, which leads to the surface of the soil being exposed and its intensive compaction. Whereas grazing areas are primarily places where the animals selectively nibble the sward, and the droppings left are more dispersed. The duration of a single accelerometer measurement (epoch time) adopted by us allows us to distinguish between grazing and resting, and due to the high probability of continuity of the activity performed, it also let to estimate the time of individual sessions (eating or resting).

We hope that our model will enable planning for monitoring large grazing areas and regulating grazing (e.g. temporarily excluding the access of animals to habitats that are excessively exploited by animals). This will also allow for purposeful, strategic grazing planning and better time management. It will be particularly important in protected areas, where the aim is not only to reduce secondary succession as part of the protection of open areas, but also to maintain the proper condition of habitats, taking into account the preservation of their species composition. Moreover, learning how stress agents such as high temperature in the summer or limited availability of forage resources in the winter will allow the animals’ behaviour-based environmental requirements to be defined. An additional benefit of such monitoring of animal activity patterns could be assessing horses’ individual health.

## Conclusion

Our research indicates the usefulness of the accelerometer and proposed artificial intelligence methods, particularly based on neural networks, in distinguishing the main levels of activity horses perform. This should facilitate the monitoring of semi-wild horses and the planning of conservation tasks for grazing plant communities. It should also be useful in research on the role of large herbivores in ecosystems.

## Data Availability

The datasets analysed during the current study are available from the corresponding author on reasonable request.

## Declarations

### A statement on the welfare of animals if the research involved animals

No approval of research ethics committees was required to accomplish the goals of this study because experimental work did not involve capturing and chemical immobilisation of the horses.
